# Successful recovery of COVID-19-associated recurrent diarrhea and gastrointestinal hemorrhage using convalescent plasma

**DOI:** 10.1186/s40779-020-00273-5

**Published:** 2020-09-23

**Authors:** Li-Bo Zhang, Rong-Rong Pang, Qing-Hua Qiao, Zhi-Hua Wang, Xin-Yi Xia, Chang-Jun Wang, Xiao-Li Xu

**Affiliations:** 1 COVID-19 Research Center, Institute of Laboratory Medicine, Jinling Hospital, Nanjing University School of Medicine, the First School of Clinical Medicine, Southern Medical University, Nanjing, 210002 Jiangsu China; 2 Department of Laboratory Medicine, Nanjing Red Cross Blood Center, Nanjing, 210003 Jiangsu China; 3 Medical and Technical Support Department, Pingdingshan Medical District, the 989th Hospital, Pingdingshan, 467000 Henan China; 4 Department of Laboratory Medicine & Blood Transfusion, Wuhan Huoshenshan Hospital, Wuhan, 430100 Hubei China; 5 Department of Laboratory Medicine & Blood Transfusion, the 907th Hospital, Nanping, 350702 Fujian China; 6 Joint Expert Group for COVID-19, Wuhan Huoshenshan Hospital, Wuhan, 430100 Hubei China; 7 Center for Disease Control and Prevention of PLA, No. 20 Dongda Street, Fengtai District, Beijing, 100071 China; 8 Department of Medical Administration, Jinling Hospital, Nanjing University School of Medicine, No. 305 East Zhongshan Road, Nanjing, 210002 Jiangsu China

**Keywords:** Coronavirus disease 2019 (COVID-19), Convalescent plasma, SARS-CoV-2 virus, Gastrointestinal symptoms

## Abstract

**Background:**

Gastrointestinal symptoms are not rare among coronavirus disease 2019 (COVID-19) patients, but there have been no reports regarding convalescent plasma therapy for the recovery of gastrointestinal problems in COVID-19 patients.

**Case presentation:**

We present two cases of patients with COVID-19-associated recurrent diarrhea and positive fecal occult blood who successfully recovered after a one-time convalescent plasma administration.

**Conclusion:**

When COVID-19 patients develop recurrent or refractory gastrointestinal symptoms and fail to respond to the available treatment, alternative therapy with convalescent plasma administration may be considered.

## Background

Coronavirus disease 2019 (COVID-19) is an emerging disease caused by a novel severe acute respiratory syndrome coronavirus 2 (SARS-CoV-2), and it has spread throughout the world [1]. In terms of clinical presentation of the new coronavirus infection, the main clinical presentation is acute febrile illness with pulmonary manifestations [2]. However, recent studies have reported the detection of the SARS-CoV-2 virus in feces [3–5], and endoscopic examination revealed COVID-19-associated ulcers and bleeding in gastrointestinal tissues in some patients [6]. In autopsy studies, the beaded intestine may be a more intuitive finding [7]. Accumulated evidence supports SARS-CoV-2 being transmitted through the gastrointestinal tract and targeting the gastrointestinal tract through angiotensin-converting enzyme 2 (ACE2) [8].

In fact, gastrointestinal symptoms are not rare in COVID-19 patients. In previous reports, 2–10% of patients with COVID-19 had gastrointestinal symptoms, such as diarrhea, vomiting and nausea [9–13]. Gastrointestinal bleeding was also found in SARS-CoV-2 infected patients [6, 14].

To date, there is no effective antiviral therapy for COVID-19. The main treatments are supportive care in the forms of supplementary oxygen therapy and extracorporeal membrane oxygenation. Convalescent plasma (CP) has been used as a last resort to treat many infectious diseases. Expectedly, CP infusion was suggested for the treatment of SARS-CoV-2 infection, and it was actually tried in several cases during the COVID-19 outbreak. Ye et al. showed a significant elimination of the virus and resolution of lung infiltrates in patients treated with CP [15]. Zhang et al. reported a severe COVID-19 patient who had an improvement in her clinical status after CP infusion in Nanjing [16]. Nevertheless, there have been no reports regarding convalescent plasma therapy for the recovery of gastrointestinal problems in COVID-19 patients. Therefore, we present two cases of patients with COVID-19-associated recurrent diarrhea and positive fecal occult blood who successfully recovered after a one-time convalescent plasma administration.

## Case presentation

### Case 1

A previously healthy 69-year-old woman was admitted to Wuhan Huoshenshan Hospital with a 20-day history of anorexia and fatigue, as well as a 7-day history of fever. Her highest temperature was 38.5 °C, accompanied by progressing fatigue and anorexia after exposure at a bank in Wuhan 1 month prior. After that, she was admitted to a local hospital and was administered a medical history of Lianhuaqingwen capsule and arbidol, with a subsequent improvement of her respiratory symptoms.

On admission, her temperature had returned to normal and the SARS-CoV-2 test of her throat swab was negative. In contrast, she presented with nausea, anorexia and loose stool, with an intermittent dry cough during the preceding 3 weeks. A routine stool check demonstrated a loose sample with positive occult blood. Routine blood, chemistry and coagulation tests showed no abnormalities. The pertinent laboratory results are summarized in Table 1. The lymphocyte subset percentages are summarized in Table 2.

**Table 1:** Serial laboratory results of the patients

Variables	Case 1					Case 2				
	HD2	HD8	HD30	HD32	HD33	HD2	HD9	HD12	HD14	HD17
White blood cell count (× 10^9^/L)	3.4	4.2	6.6	6	–	13.8	7.4	6.9	7.8	11.5
Neutrophils (%)	59	53.9	62.2	54.9	–	90	81.8	81	83.1	86.3
Lymphocytes (%)	30	37.2	25.8	34.2	–	5.4	10.4	10.9	9.5	8.5
Hemoglobin (g/L)	130	138	132	142	–	87	84	89	91	96
Platelet count (×10^9^/L)	165	179	197	181	–	188	235	222	195	245
C-reactive protein (mg/L)	0.34	0.51	1.22	1.11	–	42.4	15.1	9.59	4.81	1.99
Interleukin-6 (pg/mL)	–	–	–	3.62	–	43.6	–	–	–	–
Procalcitonin (ng/mL)	–	–	–	–	0.07	0.24	0.03	–	–	–
Albumin (g/L)	39.5	39	–	–	38.7	30.5	34.2	40.3	39	41.6
Alanine aminotransferase (IU/L)	16.7	13.4	–	–	11	8.3	15.7	12.2	9.6	7.9
Aspartate aminotransferase (IU/L)	31	29.3	–	–	23.8	11.6	8.9	8.7	8	7.2
Lactate dehydrogenase (IU/L)	201	180	–	–	192	243	187	–	163	186
α-hydroxybutyrate dehydrogenase (IU/L)	166	144	–	–	151	201	160	–	141	157
Creatine kinase (IU/L)	51.2	49.9	–	–	38	12.7	6.6	–	6.2	7.4
Cardiac troponin I (ng/mL)	0.01	0.01	–	–	–	–	–	–	–	–
Urea (mmol/L)	1.69	2.95	–	–	4.76	2.03	2.34	–	3.17	3.3
Creatinine (μmol/L)	60.9	55.2	–	–	53.2	59.5	39.3	–	38.6	39.5
Cys-C (mg/L)	0.95	1.01	–	–	0.99	1.18	1.01	–	1.13	1.23
Prothrombin time (s)	13	12.9	–	12.8	–	12.2	12.6	–	12.8	12.7
Activated partial-thromboplastin time (s)	29.3	27.6	–	28.6	–	29	24.5	–	26.5	23.6
Fibrinogen (g/L)	2.59	2.93	–	2.8	–	3.63	3.69	–	2.95	2.96
D-dimer (mg/L)	0.13	0.08	–	0.25	–	1.05	1.15	–	0.66	1.04

-. No data; *HD* Hospital day

**Table 2:** Lymphocyte subset percentages of both patients before CP infusion (on HD8 for case 1; HD7 for case 2)

Patient	CD3+ (%)	CD3 + CD4+ (%)	CD3 + CD8+ (%)	CD4/CD8 ratio	CD3-CD19+ (%)	CD3-(CD16+/CD56+) (%)
Case 1	78.40	55.20	22.10	2.5	7.00	14.40
Case 2	59.60	27.20	25.30	1.1	26.40	8.50

*CP* Convalescent plasma, *HD* Hospital day

The initial treatment was supportive and most of her symptoms improved, including cough and shortness of breath; however, after an 18-day hospitalization, she presented recurrent gastrointestinal complaints of anorexia and mild diarrhea. On hospital day (HD) 21, HD25 and HD29, the SARS-CoV-2 RNA tests of her throat swabs were positive, after two consecutively negative results on HD16 and HD19.

**Fig. 1:**
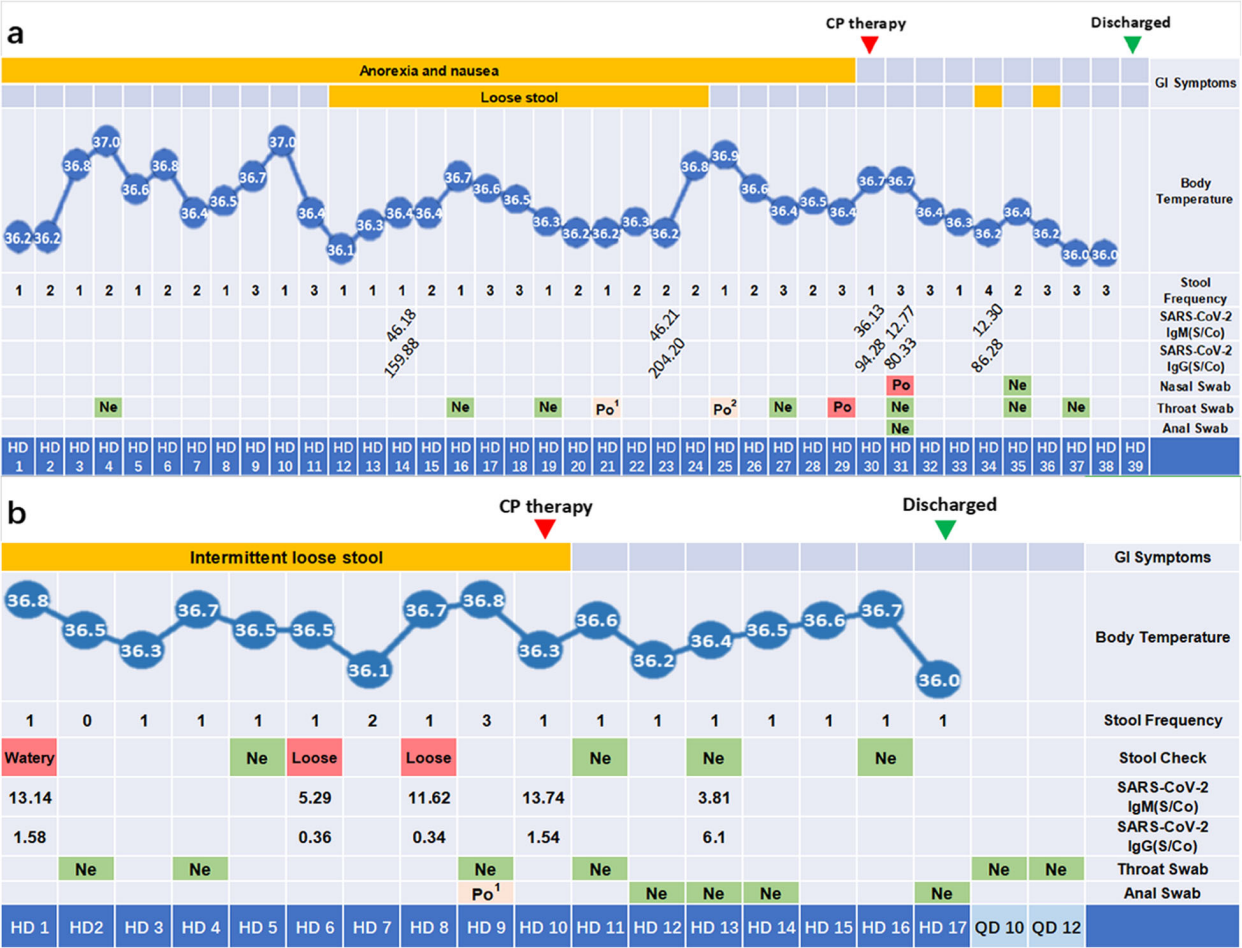
Clinical course and laboratory findings of the patients. a. Clinical course and laboratory findings of the patient in Case 1; b. Clinical course and laboratory findings of the patient in Case 2; Ne. Denotes negative; Po^1^. Positive for the nucleoprotein (N) gene region target; Po^2^. Positive for the open reading frame (ORF) target; Po. Positive for the two aforementioned targets

On HD30, approximately 400 mL of CP was administered to the patient. No adverse reaction occurred after CP infusion. On HD31, SARS-CoV-2 RNA tests were negative on her throat and anal swabs, whereas SARS-CoV-2 RNA remained detectable in her nasal swab. On HD33, the patient’s condition was much improved, with subsiding anorexia and nausea. On HD35, the SARS-CoV-2 RNA test of her nasal swab returned negative, which was consistent with her throat swab. On HD36, the patient recovered from both respiratory and gastrointestinal symptoms except for mild diarrhea. She showed complete recovery on HD38 and was discharged on HD39 (Fig. 1). SARS-CoV-2 RNA remained negative at her follow-up visits on quarantine day (QD) 6 and QD10.

### Case 2

A 50-year-old woman with a history of systemic lupus erythematosus (SLE), lacunar infarction (LI), and diabetes presented with intermittent fever, headache, dizziness and shortness of breath for 7 days. Her highest temperature was 38 °C, and she experienced chills 1 month prior. She was later treated for COVID-19 on the basis of characteristic computed tomography (CT) imaging of the thorax at another hospital in Wuhan. She received prednisone 30 mg once daily with ciclosporin, hydroxychloroquine, and total glucosides of paeony for the treatment of SLE.

At the time of arrival, the patient had recovered from dyspnea, and she maintained a normal temperature throughout the hospitalization. On examination, she was negative for the SARS-CoV-2 nucleic acid test, accompanied by a positive IgM serology finding. A routine stool check revealed a watery stool sample. Serial fecal examinations from HD1 to HD10 revealed an intermittent course of diarrhea, and she had positive fecal occult blood on HD5. A positive result of a SARS-CoV-2 RNA test from an anal swab provided the evidence of gastrointestinal infection on HD9. Additionally, a routine blood examination demonstrated a low percentage of lymphocytes (5.4%) but relatively normal levels of the most abundant subpopulations (Table 2), a low concentration of hemoglobin (87 g/L) and elevated C-reactive protein (CRP) (42.4 mg/L). Further laboratory details are summarized in Table 1.

As she had a persistent low level of anti-SARS-CoV-2 IgG antibodies, which might be due to the administration of immunosuppressors such as prednisone and ciclosporin for SLE therapy, CP transfusion was considered. On HD10, 200 mL of ABO-compatible CP with relative high titers of anti-SARS-CoV-2 antibodies (Table 3) was transfused to the patient. The stool consistency was improved immediately after CP infusion, while the fecal occult blood test turned to negative on HD13, but later turned to weak positive again on HD16. Two days after the CP infusion, serial tests from anal swabs of SARS-CoV-2 RNA were consecutively negative on HD12, HD13, HD14 and HD17, and CRP decreased to the normal range (1.99 mg/L). She showed complete recovery and was discharged on HD17, and SARS-CoV-2 RNA remained negative on QD10 and QD12 (Fig. 1).

**Table 3:** Levels of antibodies against subunits of SARS-CoV-2 from donors of CP and recipients after CP infusion

Antibodies	Case 1 Donor		Case 1 Recipient		Case 2 Donor		Case 2 Recipient	
	FI	S/Co	FI	S/Co	FI	S/Co	FI	S/Co
IgM								
S-IgM	480,724	3.251	820,248	5.547	977,929	6.614	71,465	0.483
RBD-IgM	523,293	1.951	1,825,435	6.805	1,114,862	4.156	349,693	1.304
N-IgM	295,140	1.740	105,401	0.621	844,852	4.981	222,735	1.313
IgG								
S-IgG	1,338,844	10.949	4,699,322	38.430	2,106,545	17.227	21,736	0.178
RBD-IgG	3,121,978	15.601	9,852,641	49.235	2,075,661	10.372	8946	0.045
N-IgG	1,650,382	8.038	8,326,749	40.553	3,731,186	18.171	19,841	0.097

*CP* Convalescent plasma, *FI* Fluorescent intensity, *S/Co* Signal to cut-off ratio, *IgM* Immunoglobulin M, *S-IgM* S protein specific immunoglobulin M, *RBD-IgM* Immunoglobulin M against surface spike protein receptor binding domain, *N-IgM* N protein specific immunoglobulin M, *IgG* Immunoglobulin G, *S-IgG* S protein specific immunoglobulin G, *RBD-IgG* Immunoglobulin G against surface spike protein receptor binding domain, *N-IgG* N protein specific immunoglobulin G

## Discussion and conclusions

Gastrointestinal symptoms such as diarrhea and anorexia have been widely reported in COVID-19 patients. However, this is the first report focused on the therapeutic effect of CP on COVID-19-associated gastrointestinal symptoms.

A previous study reported that COVID-19 patients with gastrointestinal symptoms were more often to have an illness duration of more than 7 days compared with those without symptoms [17]. Consistent with this finding, our patients experienced long hospital stays (39 days for Case 1 and 17 days for Case 2). A recent study revealed that all patients were tested positive for anti-SARS-CoV-2 IgG within 19 days after the symptom onset of COVID-19, and during the first 3 weeks after symptom onset, IgG and IgM antibody titers were increased [18]. From symptom onset to CP infusion, both patients exceeded the timing by a large margin (62 days for Case 1 and 42 days for Case 2). Both patients presented afebrile and were negative for SARS-CoV-2 in a throat swab test on admission, while gastrointestinal symptoms and positive anal swabs provided evidence of gastrointestinal infection. The levels of IgG antibodies, typically the antibody against surface spike protein receptor binding domain (RBD), which is an indication of neutralizing activity [19], were relatively high in both CP units. After CP infusion, the levels of antibodies against all SARS-CoV-2 subunits were elevated in one patient but were declined in the other patient (Table 3). However, fortunately, both patients recovered from their gastrointestinal symptoms and were discharged from the hospital.

The symptom of diarrhea can be aggravated by various drugs, including antibiotics, antiviral agents and certain traditional Chinese medicines, which usually cause nausea and diarrhea. Similarly, gastrointestinal hemorrhage can also be affected by many factors, such as the administration of corticosteroids and nonsteroidal antiinflammatory drugs (NSAIDs) or the development of ulcer after physiological stress. It is difficult to assess whether the gastrointestinal manifestations were primary or secondary to SARS-CoV-2 infection. Although we selected two cases with coexisting symptoms of diarrhea and gastrointestinal bleeding and considered that the symptoms were more likely a result of SARS-CoV-2, it was still challenging to trace whether the gastrointestinal abnormalities were due to SARS-CoV-2 or other factors, such as arbidol in Case 1 and prednisone administration in Case 2, which might confound the results.

Due to limitations imposed by insufficient knowledge of the virus at the beginning of the epidemic and an unprecedented demand in medical resources caused by the sudden outbreak, more data such as the presence of SARS-CoV-2 RNA in the stool is needed to strengthen our findings. However, with the increasingly improved understanding of COVID-19, a stool test or anal swab test should be taken into consideration. A previous study demonstrated that a time window might exist between respiratory and fecal specimens of PCR tests, which indicates that the virus might survive longer in the gastrointestinal tract than in the respiratory tract [20]. More formidably, in another study, Hosoda et al. reported that even after recovering from diarrhea due to COVID-19, patients could continue to excrete the virus for weeks [21]. Therefore, we suggest that a further negative nucleic acid result on an anal swab or feces test is needed before a patient is discharged from the hospital, rather than the absence of gastrointestinal symptoms. In some cases, COVID-19 patients may also present with atypical symptoms such as acute abdomen [22, 23], and it remains unclear whether COVID-19 could spread via the fecal-oral route. Given these risks, we recommend routine detection of SARS-CoV-2 for patients with a new-onset digestive symptom after a possible COVID-19 exposure.

In conclusion, we reported two cases of patients with COVID-19-associated recurrent diarrhea and positive fecal occult blood who successfully recovered after a one-time convalescent plasma administration, and we called attention to gastrointestinal infection due to COVID-19. When COVID-19 patients develop recurrent or refractory gastrointestinal symptoms and fail to respond to the available treatment, alternative therapy with convalescent plasma may be considered.

## Data Availability

Not applicable.

## Abbreviations

ACE2: Angiotensin-converting enzyme 2
COVID-19: Coronavirus disease 2019
CP: Convalescent plasma
CRP: C-reactive protein
CT: Computed tomography
HD: Hospital day
LI: Lacunar infarction
ORF: Open reading frame
QD: Quarantine day
RBD: Receptor binding domain
SLE: Systemic lupus erythematosus
